# Is *Punica granatum* Efficient Against Sepsis? A Comparative Study of Amifostine Versus Pomegranate

**DOI:** 10.3390/life15010078

**Published:** 2025-01-10

**Authors:** Kazim Sahin, Sena Sahin Aktura, Ilkay Bahceci, Tolga Mercantepe, Levent Tumkaya, Atilla Topcu, Filiz Mercantepe, Omer Faruk Duran, Huseyin Avni Uydu, Zihni Acar Yazici

**Affiliations:** 1 Department of Microbiology, Faculty of Medicine, Recep Tayyip Erdogan University, 53020 Rize, Turkey; 2 Department of Histology and Embryology, Faculty of Medicine, Recep Tayyip Erdogan University, 53020 Rize, Turkey; 3 Department of Histology and Embryology, Faculty of Medicine, Ondokuz Mayıs University, 55139 Samsun, Turkey; 4 Department of Pharmacology, Faculty of Medicine, Recep Tayyip Erdogan University, 53020 Rize, Turkey; atilla.topcu@erdogan.edu.tr; 5 Department of Endocrinology and Metabolism Diseases, Faculty of Medicine, Recep Tayyip Erdogan University, 53020 Rize, Turkey; 6 Department of Biochemistry, Faculty of Medicine, Samsun University, 55080 Samsun, Turkey

**Keywords:** amifostine, cecal-ligation puncture, lung, *Punica granatum* peel, sepsis

## Abstract

Sepsis is a clinical condition causing tissue damage as a result of infection and an exaggerated immune response. Sepsis causes 11 million deaths annually, a third of which are associated with acute lung injury (ALI). Rapid and effective treatment is crucial to improve survival rates. *Punica granatum* (pomegranate) is rich in polyphenols and demonstrates strong antioxidant activity, while amifostine acts as a free radical scavenger. This study aimed to investigate the antioxidant and anti-inflammatory effects of *P. granatum* peel extract (PGPE) and amifostine in sepsis-related ALI. Experimental groups included Control, CLP (cecal ligation and puncture-induced sepsis), Amf (200 mg/kg amifostine, intraperitoneally), and PGPE250, and PGPE500 (250 and 500 mg/kg PGPE via oral gavage, respectively). Thiobarbituric acid reactive substances (TBARS), total thiol (TT), tumor necrosis factor-alpha (TNF-α) levels, and metalloproteinases 2 and 9 (MMP-2 and MMP-9) were assessed in the lung tissue. Biochemical analysis demonstrated that TBARS and TNF-α levels significantly decreased in both the PGPE and amifostine treatment groups compared to the CLP group, while TT levels showed notable improvement. Histopathological evaluation revealed reduced MMP-2 and MMP-9 immunopositivity in the PGPE250 and PGPE500 groups. These findings highlight the lung-protective properties of PGPE, underscoring its potential as a therapeutic agent for sepsis-induced acute lung injury.

## 1. Introduction

Sepsis is an exaggerated immune response resulting from infection leading to multi-organ failure and death. Between 1990 and 2017, 48.9 million sepsis cases and 11 million sepsis-related deaths were reported globally [1]. Sepsis has a highly complex progression due to its multifaceted pathophysiology and clinical manifestations.

The cecal ligation and puncture (CLP) model is a widely used animal model of polymicrobial sepsis, where intestinal perforation facilitates the dissemination of microorganisms into the peritoneal cavity, thereby driving sepsis pathogenesis [2]. Severe diffuse fecal peritonitis commonly progresses to septic shock and multi-organ dysfunction syndrome (MODS) [3]. The lung is one of the first tissues affected by organ failure in sepsis [4]. Acute lung injury (ALI) and acute respiratory distress syndrome (ARDS) result from the disruption of the microvascular barrier due to inflammation and oxidative stress [5]. Leukocyte infiltration across the epithelial layer, pulmonary fluid accumulation, and damage to epithelial and endothelial tissue occur [6]. Lung epithelial cells secrete pro-inflammatory cytokines like tumor necrosis factor-alpha (TNF-α) and inflammatory mediators such as reactive oxygen species (ROS) [7]. Intense inflammation increases alveolar–capillary barrier permeability, leading to the accumulation of fluid in tissues and alveolar spaces. Although the underlying mechanisms have not been fully elucidated, depletion of antioxidants, lack of energy, and disruption of the oxidative balance as a result of mitochondrial dysfunction increase the ROS and lipid peroxidation rate, culminating in lung tissue damage [8]. Activation of the coagulation system and matrix metalloproteinases (MMP) in the early period in response to inflammation impairs blood circulation in tissues [9,10]. Thus, tissue damage ensues as a result of the cytokine storm [11].

Mortality due to ALI is very high, constituting one-third of sepsis-related deaths [11,12]. An important way to reduce mortality and morbidity due to sepsis is by preventing lung damage caused by sepsis. The current treatments include respiratory and fluid support, mechanical ventilation, and antibiotic provision [9]. However, widespread misuse and overuse of antibiotics have precipitated a global antimicrobial resistance problem [13]. Antimicrobial resistance highlights the need for novel therapeutic strategies.

Despite all the advances in diagnosis and treatment, there is no effective and specific treatment for ALI. Some plants and derivatives are known to have pharmacological properties, including anti-inflammatory activity. Recent research supports the medicinal use of *Punica granatum* (PG) in this context. All parts of the plant contain variable amounts of bioactive metabolites like polyphenols and flavonoids [14,15,16]. Pomegranate peel, which constitutes approximately 60% of the total fruit weight, is richer in polyphenols and flavonoids compared to fruit grains [17]. The polyphenols exert anti-inflammatory, antioxidant, antibacterial, antifungal, anticarcinogenic, antidiabetic, and anti-parasitic properties [15,16,17,18,19,20,21]. Punicalagin and ellagic acid are the main components of PG peel that exhibit strong antiproliferative activity [14]. The anti-inflammatory effects of PG have been shown in various respiratory diseases, lipopolysaccharide-induced ALI, and peritonitis [22,23,24]. Recent studies support the use of *P. granatum* as a potential source of therapeutic agent for addressing sepsis and related complications.

**Figure 1:**
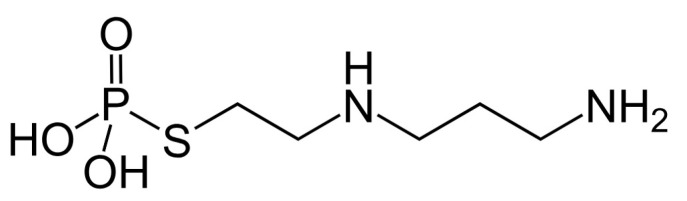
Amifostine.

Amifostine (S-2-[3-aminopropylamino]ethylphosphorothioic acid, Figure 1), also known as WR-2721, has received approval from the United States Food and Drug Administration (FDA) for specific clinical applications as a cytoprotective agent. It mitigates dose-limiting toxicities associated with cisplatin chemotherapy and ionizing radiation [25,26].

The cytoprotective effects of amifostine are attributed to its ability to scavenge free radicals and antioxidant activity. The active metabolite interacts with ROS, thereby mitigating oxidative stress. Additionally, amifostine stabilizes the cellular membrane and enhances DNA repair mechanisms, reducing the extent of injury caused by radiation or chemotherapy. Amifostine boosts endogenous protective responses by increasing intracellular glutathione levels, a critical antioxidant in maintaining redox balance [27]. Thiols are organic compounds containing a sulfhydryl (-SH) group, which plays a critical role in combating oxidative stress. Higher levels of total thiol (TT) typically correlate with increased antioxidant capacity. Thiols, due to their -SH groups, act as key antioxidants, helping to neutralize ROS and prevent oxidative damage. Inflammation can lead to oxidative stress and lipid peroxidation, subsequently, increasing thiobarbituric acid reactive substances (TBARS) levels, which correlate with the level of ROS-induced damage [28] and modulate inflammatory responses by inhibiting the production of proinflammatory cytokines, further contributing to tissue protection.

This study aimed to explore the therapeutic potential of *P. granatum* peel extract (PGPE) and amifostine in the context of sepsis-related ALI induced by CLP. Experimental groups included Control, CLP (cecal ligation and puncture-induced sepsis), Amf (200 mg/kg amifostine administered intraperitoneally), and PGPE250, and PGPE500 (250 and 500 mg/kg PGPE via oral gavage, respectively). This study assessed the TBARS, an indicator of lipid peroxidation and oxidative stress; TT, a measure of antioxidant capacity, and TNF-α, MMP-2, and MMP-9, indicators of tissue damage, in lung tissue.

## 2. Materials and Methods

### 2.1. The Experimental Animals and Study Groups

Fifty male Sprague-Dawley rats weighing 350 ± 25 g were used in this study. All animals were treated according to the principles outlined in the National Research Council Guidelines for the Care and Use of Laboratory Animals. All rats were housed in standard plastic cages on a sawdust floor under controlled lighting conditions (12 h dark: 12 h light), 55 ± 10% humidity, and 22 ± 1 °C temperature conditions. Unlimited access to standard feed and tap water was allowed. The rats with similar average weights were randomly assigned to five groups (*n* = 10 animals per group): Control, CLP, Amf, PGPE250, and PGPE500. The Control group served as the healthy group, which received 0.9% NaCl solution by oral gavage for nine days. The CLP group underwent cecal ligature and puncture to induce sepsis without drug administration [29]. The Amf group received 200 mg/kg of amifostine intraperitoneally 15 min before sepsis induction [30]. In groups PGPE250 and PGPE500, 250 and 500 mg/kg/day, *P. granatum* peel extracts were administered via oral gavage for nine days, respectively [31].

### 2.2. Cecal Ligation and Puncture (CLP)-Induced Sepsis Model

Sepsis was induced by the CLP-induced sepsis model in rats as previously described by Rittirsch et al. [29]. All surgical procedures were performed under sterile conditions. Rats were anesthetized with 100 mg/kg ketamine and 10 mg/kg xylazine injection. After the rats were tested for adequate anesthesia, a 2.5–3 cm incision was made in the midline of the abdomen. The internal organs and cecum were separated from this small incision, and the cecum was ligated with a 3/0 silk suture distal to the ileocecal valve. Similar to previous studies, two holes were drilled distal to the cecum, and the contents of the cecum were brought into contact with the peritoneum [32]. After washing the wound with 1% lidocaine for analgesia, it was closed with two layers of sterile silk 4/0 sutures. The experiment was terminated 16 h after the relevant processes were completed [33]. At the end of the experiment, the rats were euthanized by administering a high-dose anesthetic. A portion of the lung tissue was stored at −80 °C for use in biochemical studies. The other part was placed in 10% neutral formalin for histopathological and immunohistochemical analyses.

### 2.3. The Procurement and Preparation of P. granatum Peel Extract

*Punica granatum* was purchased in a public bazaar (Adiyaman, Turkey). The peel samples were dried at room temperature and then 15–20 g of peel powder was extracted with increasing methanol concentrations (50%, 80%, and 100% m/w). The doses of 250 mg/kg and 500 mg/kg of PGPE were administered to rats and prepared using an 80:20 methanol–water extraction.

### 2.4. HPLC Analyses and Quantification

The content of *P. granatum* peel was determined by high-performance liquid chromatography (HPLC). The HPLC processing was followed as described [34]. Dried plant material was ground using a laboratory mill to create a uniform powder. Approximately 0.1 g of this powder was extracted in 10 mL of methanol at concentrations of 50%, 80%, and 100% through ultrasonication at 40 °C for 60 min in an ultrasonic bath. The subsequent extracts were filtered with a 0.22 mm pore-size membrane filter (Carl Roth GmbH, Karlsruhe, Germany) and stored in a refrigerator at 4 °C until analysis. The drying and extraction processes were conducted in the dark. An RP-18 column (5 mm, 250 mm × 4.0 mm) was used with a Shimadzu LC-2030C-3D HPLC system (Shimadzu Corporation, Kyoto, Japan) equipped with a PDA detector (Shimadzu Corporation, Kyoto, Japan) to separate phenolic acids. A binary gradient elution approach was employed to identify the specific compounds. The mobile phase A was comprised of water with 0.3% phosphoric acid, while mobile phase B was water with 0.3% acetonitrile. The elution gradient was configured as follows: 10% B from 0–10 min, 25% B from 10–30 min, 60% B from 30–38 min, 60% B from 38–45 min, and returning to 10% B from 45 to 45.01 min. The analysis was conducted at a column temperature of 25 °C, with a flow rate of 0.6 mL/min and an injection volume of 10 µL. Calibration of the components was performed at 203–280–320–360 nm using standard solutions at concentrations of 5, 10, 20, 50, 100, and 200 ppm. The HPLC analysis results of pomegranate peel extract are detailed and exhibit a profile that aligns with literature data [35].

### 2.5. Biochemical Analysis

#### 2.5.1. Tissue Sampling and Homogenization

The homogenization buffer (pH 7.4) was prepared with 20 mM sodium phosphate + 140 mM potassium chloride. Then, 1 mL of homogenization buffer was added to 0.1 g of lung tissue and homogenized in the Qiagen TissueLyser LT device (Qiagen, Venlo, The Netherlands) [36]. After homogenization, the sample was centrifuged at 800× *g* for 10 min at 4 °C. The TBARS, TT, and TNF-α levels were determined in the supernatant.

#### 2.5.2. Determination of TBARS

TBARS determination was performed according to the method described [37]. In brief, 200 µL lung tissue supernatant, 375 µL of 20% acetic acid (*v*/*v*) (pH 3.5), 375 µL of 0.8% thiobarbituric acid (TBA), and 50 µL of 8.1% sodium dodecyl sulfate (SDS) were mixed, vortexed, and the reaction was incubated in a boiling water bath for 1 h. After incubation, the samples were cooled in ice water for 5 min and centrifuged at 750× *g* for 10 min. The resulting pink color was determined spectrophotometrically at 532 nm. The results are expressed as nmol/g tissue.

#### 2.5.3. Determination of Total Thiol (TT)

Sedlak and Lindsay’s spectrophotometric method was used to determine the total thiol groups [38]. The –SH groups were measured using the Ellman reagent. After adding 1000 µL of 3 M Na_2_HPO_4_ and 250 µL of DTNB (4 mg DTNB was prepared in 10 mL of 1% sodium citrate solution) to 250 µL of supernatant and vortexing, the absorbance of the samples was determined at 412 nm. The results were determined by the 1000–62.5 µM reduced glutathione standard graph and are given as µmol/g tissue.

#### 2.5.4. Tissue TNF-α Determination

Tissue TNF-α levels were measured using a rat TNF-α ELISA kit (Cat. No: E0764Ra; Bioassay Technology Laboratory (BT Lab) Inc. Shanghai, China) using the sandwich immunoassay method. Results are given as ng/mL.

### 2.6. Histopathological Analysis

Lung tissue samples extracted from rats were fixed in a 10% phosphate-buffered formalin (Sigma–Aldrich, St. Louis, MO, USA solution for 36 h. Following the fixation process, routine histological follow-up procedures were performed, followed by dehydration (with increasing ethanol series, Merck GmbH, Darmstadt, Germany), mordanting (xylol, Merck GmbH, Darmstadt, Germany), embedding in soft paraffin (Merck GmbH, Darmstadt, Germany) and the final hard paraffin blocking (Merck GmbH, Darmstadt, Germany) was applied. Then, 4–45 µm thick sections were cut from the paraffin blocks with a rotary microtome (Leica RM2525, Lecia, Wetzlar, Germany) and stained with Harris hematoxylin and eosin G (H&E; Merck, GmbH, Darmstadt, Germany).

### 2.7. Immunohistochemical (IHC) Analysis

Anti-MMP-2 (rabbit polyclonal, ab86607, Abcam, UK) and anti-MMP-9 (rabbit polyclonal, ab76003, Abcam, UK) primary antibodies together with secondary antibodies (Goat Anti-Rabbit IgG H&L (HRP), ab205718, Abcam, UK) were used to determine the fibrosis in the interstitial areas and the increase in the extracellular matrix in the rat lung tissue. Sections were incubated with primary and secondary antibodies for 60 min after applying the antigen retrieval procedure using the Leica IHC/ISH (Leica Biosystems, Leica, Germany) device after deparaffinization following the manufacturer’s guidelines. Sections were stained with diaminobenzidine tetrahydrochloride (LeicaltraviewDAB, Leica Biosystems, Wetzlar, Germany) and Harris hematoxylin (Merck, Darmstadt, Germany).

### 2.8. Semi-Quantitative Analysis

Alveolar and interstitial neutrophil deposition in the lung, hyaline membranes, alveolar debris deposition, and alveolar septal wall thickness were calculated using the lung tissue damage score (LDS) of Matute-Bello et al., as shown in Table 1 [39]. In each section of lung tissue, 35 different areas were evaluated by two histopathologists blinded to the study groups. MMP-2 and MMP-9 positivity were analyzed by two blinded histopathologists (Table 2). Thirty-five different areas were selected randomly in each preparation, and IHC positivity was measured in twenty-one different areas in each group.

### 2.9. Statistical Analysis

The statistical analyses were conducted using the SPSS 18.0 (IBM, Armonk, NJ, USA) statistical program. The data were tested for a normal distribution with the Kolmogrov–Smirnov test. If the data did not follow a normal distribution, the Kruskal–Wallis test was used for statistical testing. Differences between groups were determined using the Mann–Whitney U post hoc test. The values are expressed as median. A *p*-value of <0.05 was accepted as significant.

## 3. Results

### 3.1. HPLC Results

The phenolic composition of *P. granatum* varied significantly between the inner fruit coat and the fruit peel, as well as among different methanol concentrations (50%, 80%, and 100%). The amounts were calculated in µg/g equivalent value and given in Table 3.

PUN A and B were the most abundant compounds. In the fruit coat, PUN A reached 4528.03 µg/g at 50% methanol, which was approximately 3.12 times higher than its concentration at 100% methanol (1345.47 µg/g). Similarly, PUN B in the fruit coat was 2591.47 µg/g at 50% methanol, approximately 4.05 times higher than the 639.11 µg/g observed at 100% methanol.

In the fruit coat, the EGCG concentration peaked at 274.90 µg/g with 80% methanol, which was higher than the 186.88 µg/g observed at 50% methanol.

The amount of EA was higher in the peel with 70.97 µg/g compared to the inner fruit coat with 17.84 µg/g in 80% methanol. The concentration ratio was found to be approximately 4:1 between the two sections.

The concentrations of GA, FA, Q, p-CA, CA, DHBA, CH, and UA compounds were observed to be quite modest. In contrast, CAF and CGA were detected at relatively higher levels.

### 3.2. Biochemical Results

The TBARS level in the Control group (27 ± 5 nmol/g tissue) increased to 67 ± 9 nmol/g tissue in the CLP group. In the PGPE250 group, the TBARS level was reduced to 32 ± 8, and in the PGPE500 group, to 30 ± 10 nmol/g tissue. The intense decrement in the Amf group reduced the TBARS level to 23 ± 4 nmol/g tissue. The TBARS levels revealed statistically significant differences between the CLP and other groups (*p* < 0.001, Table 4). There was also a difference between the Control group and the Amf group, as well as the PGPE250 and PGPE500 groups, but this difference was not statistically significant (*p* > 0.05, Table 4).

The TT level of the CLP group was significantly higher than the other groups (*p* = 0.001, Table 4). The TT levels of the PGPE500 group were higher than the PGPE250 and Amf groups, but the difference between the groups was not statistically significant (*p* > 0.05, Table 4). In the Amf group, TT levels were reduced to close to the Control group value.

The TNF-α levels were measured in supernatants prepared using 10 mL of buffer per gram of tissue. Results were converted from ng/g tissue to ng/mL and are presented in Table 4. The levels were statistically significantly lower in the other groups compared to the Control group (*p* < 0.05, Table 4). The TNF-α level in the Control group (20.3 ± 3.0 ng/mL) increased to 22.9 ± 1.3 ng/mL tissue in the CLP group. In the Amf group, the TNF-α levels were close to the PGPE500 group. However, the difference between groups was not statistically significant.

### 3.3. Histopathological Findings

**Figure 2:**
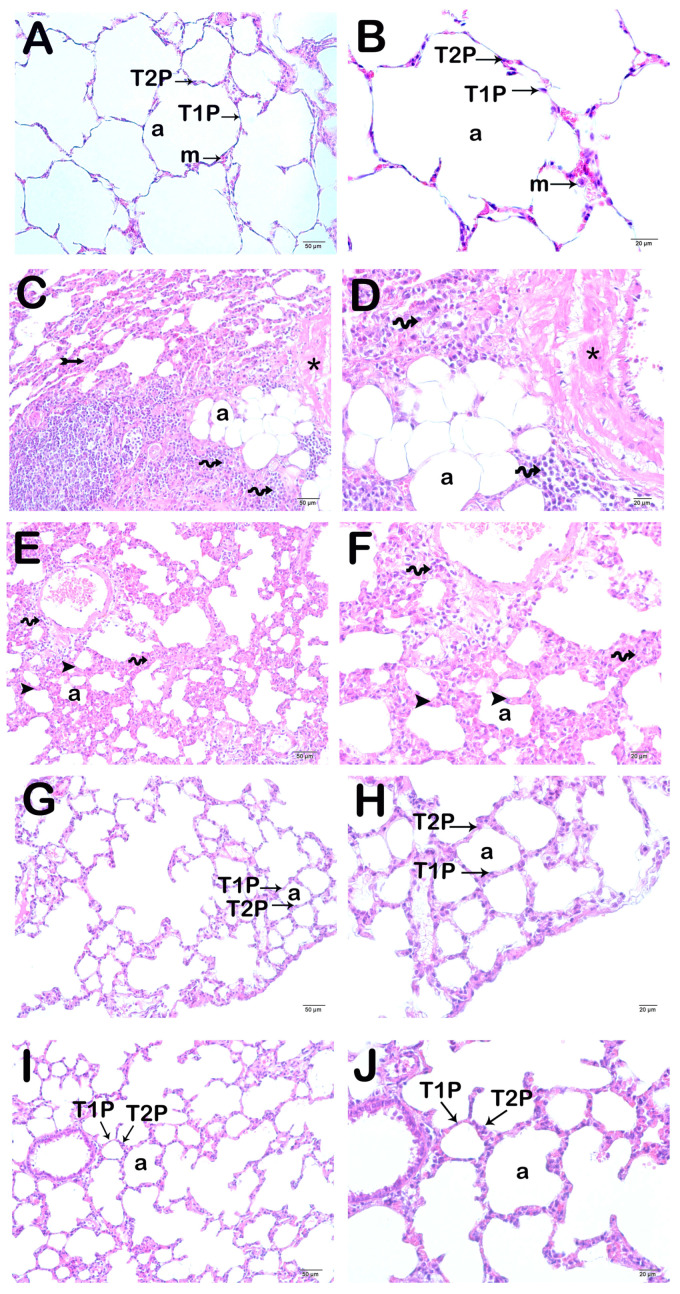
Representative light microscopy images of lung tissue sections stained with H&E. All groups consisted of 10 animals (*n* = 10), including Control, CLP, Amf, PGPE250, and PGPE500. Alveolus (a), Type I pneumocytes (thin arrow), Type II pneumocytes (thin arrow). (**A**,**B**) Control group lung tissue sections showing typical Type I pneumocytes (thin arrow) and Type II pneumocytes (thin arrow) in a normal alveolar structure (LHDS median: 1 (1-1)). A: ×20, B: ×40. (**C**,**D**) Inflammation (tailed arrow) is observed in diffuse alveolar and interstitial areas in lung tissue sections belonging to the CLP group (spiral arrow). Hyaline membrane formations (*) and alveolar septal wall thickening (arrowhead) are observed (LHDS median: 8 (8-9)). C: ×20, D: ×40. (**E**,**F**) Amf group shows decreased inflammation in alveolar and interstitial areas (spiral arrow) and decreased hyaline membrane structures and alveolar wall thickness (arrowhead) (LHDS median: 3 (2-4)). E: ×20, F: ×40. (**G**,**H**) Lung tissue sections of the PGPE250 group show decreased alveolar septal wall thickness and hyaline membrane structures. (LHDS median: 2 (1-2)). G: ×20, H: ×40. (**I**,**J**) PGPE500 group showing normal structure Type 1 (thin arrow) and Type 2 pneumocytes (thin arrow) and decrement in alveolar septal wall thickness (LHDS median: 1 (1-2)). I: ×20, J: ×40.

Type I and Type II pneumocytes in the alveolar sac were normal in the lung tissue of the Control group (Figure 2A,B, Table 5, LHDS: 1 (1-1). Diffuse inflammation in the alveolar and interstitial areas, as well as the alveolar septal wall in the lung tissue sections of the CLP group, was observed. In addition, hyaline membrane structures were present, and septal wall thickness increased (Figure 2C,D; Table 5, LHDS: 8 (8-9)). Lung tissue sections of the Amf group exhibited a decrease in alveolar and interstitial inflammation and alveolar septal wall thickness (Figure 2E,F; Table 5, LHDS: 3 (2-4)). In the PGPE250 and PGPE500 groups, alveolar and interstitial inflammation and alveolar septal wall thickness also decreased. In addition, normal Type I and Type II pneumocytes were seen in alveolar sacs in respiratory bronchioles in both groups (Figure 2G,H; Table 5, LHDS: 2 (1-2), Figure 2I,J; Table 5, LHDS: 1 (1-2), respectively).

### 3.4. Immuno-Histochemical Findings

**Figure 3:**
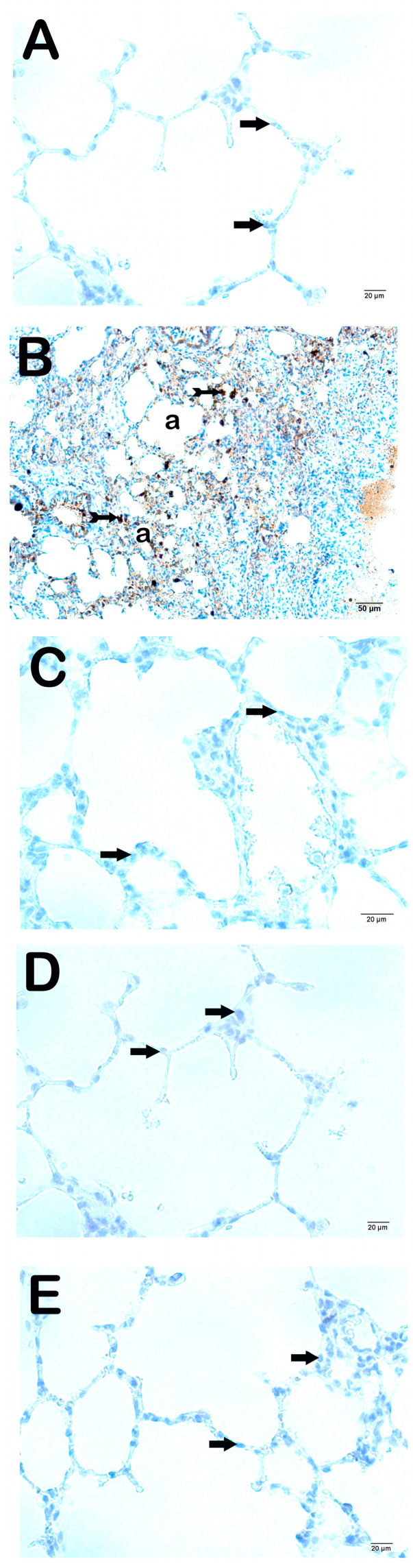
Representative light microscopy images of lung tissue sections stained with anti-MMP-2 primary antibody. All groups consisted of 10 animals (*n* = 10), including Control, CLP, Amf, PGPE250, and PGPE500. (**A**) Control group lung tissue sections showing normal Type I pneumocytes (arrow) and Type II pneumocytes (arrow) in normal structures are observed in alveolar sacs in lung tissue sections. In addition, it is observed that the interstitial areas are MMP-2 immune-negative (MMP-2 positivity score: 0 (0-0) (×40). (**B**) Sections of the CLP group showing intense MMP-2 positivity in Type I (tailed arrow) and Type II pneumocytes (tailed arrow) in the alveolar (a) sacs (MMP-2 positivity score: 2 (2-3)) (×40). (**C**) A decrease was seen in the number of MMP-2 immune-positive cells in the Amf group, while commonly immune-negative typical Type I (arrow) and Type II pneumocytes (arrow) are observed (MMP-2 positivity score: 0 (0-1)) (×40). (**D**) In the PGPE250 group, MMP-2 immune-negative Type I (arrow) and Type II pneumocytes (MMP-2 positivity score median: 0 (0-1)) (×40). (**E**) In the PGPE500 group, Type I (arrow) and Type II pneumocytes (arrow) with immuno-negative typical structure (MMP-2 positivity score: 0 (0-0)) (×40).

**Figure 4:**
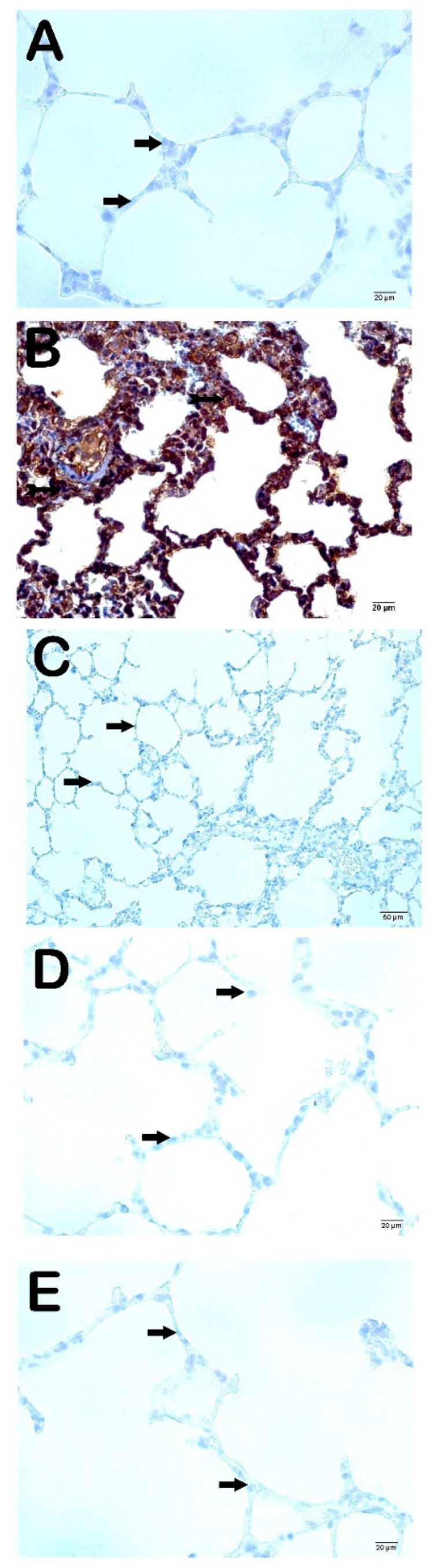
Representative light microscopy images of lung tissue sections stained with anti-MMP-9 primary antibody. All groups consisted of 10 animals (*n* = 10), including Control, CLP, Amf, PGPE250, and PGPE500. (**A**) Control group lung sections showing normal Type I (arrow) and Type II pneumocytes (arrow). In addition, the interstitial areas are MMP-9 immune-negative (MMP-9 positivity score: 0 (0-0) (×40). (**B**) CLP group sections demonstrate intense MMP-9 positivity (MMP-9 positivity score: 3 (2-3)) (×40). (**C**) Type I (arrow) and Type II pneumocytes (arrow) of the Amf group showing MMP-9 positivity in alveolar sacs are observed to be decreased in number (MMP-9 positivity score: 1 (0-1)) (×40). (**D**) In the PGPE250 group, Type I (arrow) and Type II pneumocytes with decreased immunopositivity (MMP-9 positivity score median: 0 (0-1)) (×40). (**E**) PGPE500 group showing Type I (arrow) and Type II pneumocytes (arrow) staining, but MMP-9 immunonegative in alveolar sacs (MMP-96 positivity score: 0 (0-0)) (×40).

The fibrosis in the interstitial areas and the increment in the extracellular matrix were analyzed with the IHC method; we observed that the cells showing intense immunopositivity in the CLP group was significantly increased compared to the Control group (Figure 3A and Figure 4A; Table 6; *p* = 0.001; *p* = 0.001; respectively). On the other hand, the cells showing MMP2- and MMP-9 positivity in the Amf group were significantly reduced compared to the CLP group (Figure 3B and Figure 4B; Table 6; *p* = 0.001; *p* = 0.001; respectively). Similarly, MMP2- and MMP-9 positivity significantly decreased in the PGPE250 and PGPE500 groups compared to the CLP group (Figure 3C and Figure 4C; Table 6; *p* = 0.001; *p* = 0.001; respectively; Figure 3D and Figure 4D; Table 6; *p* = 0.001; *p* = 0.001; respectively).

### 3.5. Semi-Quantitative Results

The alveolar septal wall thickness, which was 10.14 ± 2.14 µm in the Control group, increased to 28.41 ± 7.88 µm in the CLP group (Figure 2A–D; Table 7; *p* = 0.001). However, it decreased to 15.04 ± 6.12 µm in the Amf group (Figure 2E,F; Table 7; *p* = 0.001) and to 12.47 ± 2.85 µm in the PGPE250 group (Figure 2G,H; Table 7; *p* = 0.001) and to 11.89 ± 3.87 µm in the PGPE500 group (Figure 2I,J; Table 7; *p* = 0.001). Alveolar septal wall thicknesses in all groups treated with amifostine and *P. granatum* peel extract were significantly reduced compared to the CLP group and were similar to the Control group.

## 4. Discussion

The CLP-induced sepsis model was examined in this study by assessing the pro-inflammatory cytokine TNF-α, antioxidant levels, lipid peroxidation rate, and MMPs. The parameters were compared to a group administered amifostine, a thiol compound. Amifostine neutralizes ROS and reduces oxidative stress, which converts to active thiol compounds in tissue [29,40]. In this study, amifostine was used as a positive control to compare with the efficacy of PGPE, given its established antioxidant properties. Amifostine’s radioprotective, chemoprotective, and cytoprotective efficacies are well-documented [32,41,42]. Its cytoprotective effect has been attributed to DNA repair, scavenging of free radicals, and modulation of NF-kB [32]. The demonstration that amifostine reduces intra-alveolar inflammation and free oxygen radicals in ventilator-associated lung injury suggests that it will also protect against lung injury due to sepsis [36,40]. Our results showed that administering a single dose of amifostine as a pretreatment decreased TNF-α and TBARS levels and increased total thiol levels in sepsis associated with ALI. Amifostine at a dose of 200 mg/kg is within the lower end of the clinical dose range and has been shown to afford protective effects without significant adverse effects [43]. Recent clinical investigations have explored the efficacy of lower doses of amifostine, recognizing its ability to provide therapeutic benefits in various oxidative stress-related conditions, including cancer therapy and organ protection. This dosage was an appropriate and well-tolerated dose for comparison to a natural compound like PGPE. Although amifostine has demonstrated beneficial effects, its clinical application is limited due to potential side effects such as nausea, hypotension, and hypersensitivity reactions [25]. In contrast, several natural compounds, including curcumin, resveratrol, and quercetin, have shown therapeutic potential in reducing inflammation and oxidative stress in models of ARDS [41,44,45]. These compounds exert their effects by targeting key molecular pathways, such as NF-κB, TNF-α, and HDAC3, which are upregulated during LPS-induced ARDS and drive the inflammatory response. Additionally, natural products with potent antioxidant properties have been found to activate protective pathways, such as NRF2 and SOD, which play crucial roles in mitigating oxidative damage. Among these, *P. granatum* emerges as a promising candidate due to its accessibility, cost-effectiveness, and fewer anticipated adverse effects, offering a natural alternative with significant therapeutic potential.

Pomegranate is a fruit that has come to the forefront with its antioxidant and anti-inflammatory properties throughout history and has been studied extensively [19,46]. In a study comparing the antioxidant contents of the peels of pomegranate, grape, apple, and mocha fruits, the highest antioxidant activity and phenolic content were found in the PG peel [47]. Literature studies with various parts of pomegranate show that the peel has richer polyphenolic content and higher antioxidant capacity than the fruit grains [35]. In addition, the skin constitutes about half of the total fruit volume, and the peel has been evaluated as an antioxidant and cytoprotective agent [47]. The natural polyphenols in PGPE, such as tannins and flavonoids, contribute to its potent antioxidant and anti-inflammatory effects. 

In a previous study, we investigated the PGPE for its potential to mitigate inflammation and oxidative damage in the respective organ systems in the setting of LPS-induced sepsis-associated acute kidney injury [48]. Similarly, this study demonstrated a reduction in TBARS and total thiol levels in lung tissue, indicating PGPE’s lung-protective effects against oxidative damage. Furthermore, both studies utilized dose-dependent experimental groups to assess the efficacy of PGPE, revealing more beneficial effects at higher doses [44]. The doses of PGPE at 250 mg/kg and 500 mg/kg were referred from a study that effectively modulated oxidative stress markers, including superoxide dismutase (SOD) activity, glutathione (GSH) levels, and malondialdehyde (MDA) levels, as well as cytokine expression in diabetic rats. The 250 mg/kg dose was sufficient to modulate oxidative stress, a reduction in MDA levels, and increased antioxidant activity, while the 500 mg/kg dose enhanced these effects further. For instance, in a study examining the effects of PGPE on liver damage induced by oxidative stress, similar doses were found to improve antioxidant status and reduce inflammation markers [49].

The CLP-induced sepsis model is based on its well-documented ability to closely mimic human sepsis, particularly in terms of systemic inflammation, multi-organ dysfunction, and mortality. The model induces a polymicrobial infection that results in a septic response, providing a more clinically relevant representation of sepsis compared to simpler models [2]. Studies in the literature show that pomegranate peel extract also has an antimicrobial effect [47]. It has been reported that the methanolic extract of pomegranate peel shows antimicrobial properties and significantly reduces bacterial biofilm formation [47,50]. In an in vitro study investigating the antimicrobial activity of plant extracts, the antimicrobial effect of pomegranate peel extract on *A. baumannii* was demonstrated [48].

The lungs are the first organ to be affected and are the most vulnerable in sepsis [10,24]. Sepsis-related ALI is characterized by tissue damage resulting from intense neutrophil infiltration into the epithelium, accumulation of exudate in the alveoli and hyaline membranes, and loss of alveoli capillary membrane integrity [11]. This process is thought to occur due to the increase in the release of cytokines such as TNF-α from leukocytes and macrophages [31]. ALI due to sepsis is more severe and fatal than acute lung injury due to pneumonia [47]. In addition, inflammation is more challenging to resolve in sepsis-related lung injury [14]. This is probably due to the complex and multifactorial mechanism of sepsis. Although the underlying mechanism of acute lung injury due to sepsis is not fully understood, severe inflammation and oxidative stress resulting from the increase of cytokines and deterioration of the microvascular structure of the lung are the leading causes [7]. Excessive pro-inflammatory cytokine release leads to uncontrolled systemic inflammation, pathological changes, and organ dysfunction [14,51]. Therefore, the suppression of inflammation and reduction of oxidative stress should be the primary goals to prevent sepsis-related lung injury [16,50]. This study showed that pomegranate peel extract protected lung tissue through antioxidant and anti-inflammatory effects in acute lung injury associated with sepsis. This result is compatible with the literature data [24,25,31]. The increase in alveolar septum thickness and hyaline membranes accompanying the inflammation in the alveolar and interstitial areas, which we observed in the histopathological examination of the lung tissue of the sepsis group, supports the correct functioning of our model.

The key indicator of oxidative stress is TBARS, the levels in PGPE500 reduced to 30 ± 10 nmol/g tissue, closely approaching the reduction observed with amifostine (23 ± 4 nmol/g) and showing substantial improvement compared to the CLP group (67 ± 9 nmol/g, *p* < 0.001). When compared to amifostine, a synthetic thiol-based antioxidant, PGPE demonstrated comparable efficacy in reducing oxidative stress markers.

Amifostine restored TT levels as an antioxidant to near-control values (11.6 ± 1.46 μmol/g tissue) while PGPE500 achieved 16.9 ± 3.6 μmol/g tissue, which was higher than PGPE250 (13.5 ± 5.2 μmol/g tissue) and closer to the elevated levels observed in the CLP group (17.5 ± 3.1 μmol/g tissue). These results suggest that while PGPE may not completely normalize TT levels, it provides significant antioxidant support.

TNF-α levels were relatively stable across groups, with PGPE500 (2.38 ± 0.22 pg/mL) and amifostine (2.37 ± 0.53 pg/mL) showing no significant deviation from the CLP group (2.29 ± 0.13 pg/mL). These findings imply that while both treatments effectively mitigate oxidative stress, their impact on inflammation, as measured by TNF-α, may require further investigation, potentially focusing on alternative inflammatory markers. In these studies, similar to our study, pomegranate peel inhibited TNF-α production and reduced neutrophil migration [25,31]. In our study, we think that pomegranate peel extract and amifostine have a protective and curative effect in sepsis-related lung injury by decreasing the release of pro-inflammatory cytokines, increasing the level of antioxidants and lowering the levels of MMPs responsible for the damage. Decreased TBARS and TNF-α levels and increased total thiol levels in the lung tissue of CLP-induced sepsis rats are consistent with the literature data, showing that our sepsis model works and that sepsis-related lung damage has occurred [33,52]. Batcik et al. demonstrated a similar efficacy of amifostine on CLP-induced acute kidney injury (AKI). This overlap suggests a shared mechanism by which amifostine modulates oxidative and inflammatory pathways across different organ systems [32]. Ugan et al. showed that PGPE at 250 mg/kg and 500 mg/kg provided significant protection against sepsis-induced lung injury, as indicated by a reduction in MDA levels and an increase in antioxidant activity. Their study found that both doses effectively modulated oxidative stress markers, including superoxide dismutase (SOD) activity, glutathione (GSH) levels, and malondialdehyde (MDA) levels, as well as cytokine expression (TNF-α and IL-1β) in diabetic rats [31]. Additionally, 250 mg/kg of PGPE demonstrated a strong antihyperglycemic effect [53].

TNF-α increases the expression of adhesion molecules on endothelial cell surfaces [25]. Thus, leukocytes migrate to the alveoli and cause inflammation [10]. The decreased inflammation in the lung alveoli of the groups in which pomegranate peel extract was applied in our histopathological examination can be explained by the suppression of cytokine levels by the pomegranate peel extract. At the same time, total thiol levels and oxidant/antioxidant status indicators increased in the groups treated with pomegranate peel extract and almost approached those of the Control group. Although there was no statistically significant difference, the protective effect seemed to increase as the dose of pomegranate peel extract increased. The histopathological analysis showed that alveolar and interstitial inflammation were significantly reduced in the groups treated with pomegranate peel at 500 mg/kg dose (no statistical difference), the alveolar septum thickness was found to be similar to the Control group, and hyaline membranes were not observed. These findings are consistent with the results of studies in which pomegranate peel was used as a therapeutic agent in LPS-induced sepsis-related lung injury and LPS-induced lung injury in diabetic rats [24,31]. Pomegranate has been used as a therapeutic agent in respiratory diseases such as asthma, lung cancer, interstitial pulmonary fibrosis, and chronic obstructive pulmonary disease (COPD) [30]. In these studies, the protective properties of pomegranate were attributed to its anti-inflammatory and antioxidant effects. Indeed, the increase in TT accompanying the decrease in TNF-a levels in the groups treated with pomegranate peel supports these effects.

Matrix metalloproteinases (MMPs) are enzymes that degrade extracellular matrix proteins and play an important role in lung diseases [13]. A few studies suggest that MMP-2 is derived from bronchial and alveolar epithelial cells and fibroblasts. MMP-9 is released from lymphocytes, neutrophils, and alveolar macrophages [54]. The extracellular matrix-degrading effects of MMPs suggest that they play an important role in lung regeneration and fibrosis as an early response to injury [12]. High levels of MMP-2 and MMP-9 in diseases with lung fibrosis and the progression of fibrosis in mice with MMP-2 deficiency are literature data supporting this idea [54]. MMPs also play an important role in sepsis by regulating the release and activity of cytokines, initiating the inflammatory response of leukocytes. It has also been shown that MMP-9 activates TNF-α in its inactive form [13]. It is known that MMP-2 and MMP-9 levels increase in sepsis and show a positive correlation with the severity of the disease [55,56]. It has been found that MMP-9 levels are associated with disease activity and mortality in patients with COVID-19 disease hospitalized in the intensive care unit [57]. Zheng et al. observed that MMP-2 and MMP-9 activity increased in acute lung injury in mice with LPS [9]. In our study, immunohistochemical analysis of the lung tissue of rats in the sepsis group showed significantly higher MMP-2 and MMP-9 positivity than the other 4 groups. These results were consistent with those reported by Ates et al. and Ganguly et al. [4,58]. In our study, while MMP-2 positivity was not observed for both doses of pomegranate peel extract and the amifostine groups, mild MMP-9 positivity was observed only in the amifostine group. No MMP-9 positivity was observed in the groups treated with pomegranate peel extract. The literature has different implications regarding MMPs, whose levels increase in pathological processes [13,54,55]. The data indicate that increased MMP levels exacerbate sepsis and inhibition of its release has a protective effect, as well as that increased MMP is a defense mechanism against sepsis and its destruction exacerbates sepsis and sepsis-related death [12,13,54,55,56]. The decrease in MMP concentration in the groups treated with pomegranate peel and amifostine may be due to the reduction of lung damage due to the protective effect of both agents.

## 5. Conclusions

Pomegranate peel extract showed antioxidant and anti-inflammatory activity in acute lung injury due to sepsis comparable to amifostine. Therefore, this offers a suitable natural alternative to amifostine. However, some limitations should be considered. This study was conducted on an animal sepsis model that requires clinical studies to confirm the findings. Sepsis is often not a predictable clinical condition, and it is often unpredictable as to which sepsis patients will develop acute lung injury. Since the lungs are the first organ affected in sepsis, lung injury was evaluated in this study’s acute phase of sepsis. It is unknown whether the protective effects of pomegranate peel extract and amifostine will continue in chronic processes and whether they can heal chronic damage. In addition, microbiological and molecular analyses could increase our knowledge about the properties of the antimicrobial mechanism of *P. granatum* peel extract.

## Figures and Tables

**Table 1:** Lung Damage Score (LDS) modified from Matute-Bello et al.

Findings	Score		
	0	1	2
Alveolar inflammation	≤5%	≤50%	≥50%
Interstitial inflammation	≤5%	≤50%	≥50%
Arterial hyaline membrane formation	≤5%	≤50%	≥50%
Alveolar wall thickness (Treatment/Control Group)	˂×	2×–4×	>×4

**Table 2:** Immunopositivity Scoring Methods Results: median (25–75% interquartile range).

Score	Findings
0	<5%
1	<5–25%
2	<26–50%
3	>51%

**Table 3:** Phenolic Compounds of *P. granatum*.

Component	Inner Fruit Coat				Fruit Coat	
	% Concentration (µg/g)					
	50	80	100	50	80	100
Ellagic acid (EA)	17.762	17.844	17.853	46.385	70.971	42.306
Gallic acid (GA)	1.653	1.262	1.280	2.894	1.804	1.519
Ferulic acid (FA)	2.532	2.718	2.876	4.427	4.075	1.726
Quercetin (Q)	0.803	0.775	0.801	0.789	0.818	0.812
*p*-Coumaric acid (*p*-CA)	0.1	0.001	0.003	0.192	0.175	0.101
Caffeic acid (CA)	1.839	1.301	1.851	1.586	1.681	0.773
2,4-dihydroxybenzoic acid (2,4-DHBA)	3.587	2.583	7.435	11.193	11.538	9.934
Epigallocatechin gallate (EGCG)	175.950	72.864	162.763	186.88	274.901	191.394
Catechin hydrate (CH)	2.218	1.569	2.354	5.674	5.488	5.399
Caffeine (CAF)	16.01	14.321	8.972	16.328	21.708	7.547
Chlorogenic acid (CGA)	59.2	29.108	29.984	60.394	28.480	21.575
Ursolic acid (UA)	3.36	2.28	10.499	2.44	3.380	10.719
Punicalagin (A) (PUN)	4082.2165	1395.002	1179.532	4528.030	1451.663	1345.471
Punicalagin (B) (PUN)	2448.852	835.247	539.311	2591.466	827.183	639.108

**Table 4:** Biochemical Analysis Results (mean ± standard deviation).

Groups	TBARS (nmol/g Tissue)	TT (µmol/g Tissue)	TNF-α (ng/mL)
Control	27 ± 5	11.4 ± 1.7 ^a,^***	20.3 ± 3.0 ^a,^*^,b,^**
CLP	67 ± 9 ^c,^***	17.5 ± 3.1 ^d,^*	22.9 ± 1.3
Amf	23 ± 4	11.6 ± 1.46	23.7 ± 5.3
PGPE250	32 ± 8	13.5 ± 5.2	24.0 ± 3.7
PGPE500	30 ± 10	16.9 ± 3.6	23.8 ± 2.2

*: *p* < 0.01, **: *p* < 0.05, ***: *p* < 0.001. ^a^: statistically significant difference of the Control group compared with the CLP group. ^b^: statistically significant difference of the Control group compared with the PGPE250 and PGPE500 groups. ^c^: statistically significant difference of the CLP group compared with the other groups. ^d^: statistically significant difference of the CLP group compared with the Amf and PGPE250 groups.

**Table 5:** Lung Histopathological Damage Score (LHDS) Results (median 25–75% interquartile range).

Groups	Alveolar Inflammation	Interstitial Inflammation	Hyaline Membrane	Alveolar Septum Thickness (Matute-Bello et al.)	LHDS
Control	0 (0-0)	0 (0-0.5)	0 (0-0)	1 (1-1)	1 (1-1)
CLP	2 (1-2) ^a^	2 (2-3) ^a^	2 (2-2) ^a^	2 (2-3) ^a^	8 (8-9) ^a^
Amf	0 (0-1) ^b,c^	1 (1-1) ^a,b^	1 (0-1) ^a,c^	1 (1-1) ^c^	3 (2-4) ^a,b^
PGPE250	0 (0-1) ^c^	0 (0-1) ^b,d^	0 (0-0) ^c,e^	1 (1-1) ^c^	2 (1-2) ^b,d^
PGPE500	0 (0-0) ^c^	0 (0-0) ^b,d^	0 (0-0) ^c,d^	1 (1-1)^c^	1 (1-2) ^b,d^

^a^ *p* = 0.001 compared with the Control group, ^b^ *p* = 0.015 compared with the Control group, ^c^ *p* = 0.001 compared with the CLP group, ^d^ *p* = 0.001 compared with the Amf group, ^e^ *p* = 0.005 compared with the Amf group, Kruskal–Wallis-Tamhane’s T2 test.

**Table 6:** IHC Positivity Grade Score Results (median with interquartile range).

Groups	MMP-2 Positivity Score	MMP-9 Positivity Score
**Control**	0 (0-0)	0 (0-0)
**CLP**	2 (2-3) ^a^	3 (2-3) ^a^
**Amf**	0 (0-1) ^b^	1 (0-1) ^b^
**PGPE250**	0 (0-1) ^b^	0 (0-1) ^b^
**PGPE500**	0 (0-0) ^b^	0 (0-0) ^b^

^a^ *p* = 0.001 versus the Control group, ^b^ *p* = 0.001 versus the CLP group, Kruskal–Wallis-Tamhane’s T2 test.

**Table 7:** Alveolar wall thickness (µm) quantitative results (mean ± standard deviation).

Groups	Alveolar Wall Thickness	Alveolar Septum Thickness (Treatment/Control Group)	Matute-Bello et al. Modified Alveolar Septum Thickness Score
Control	10.14 ± 2.14	1.00	1 (˂X2)
CLP	28.41 ± 7.88 ^a^	2.8	2 (≥X2)
Amf	15.04 ± 6.12 ^b^	1.32	1 (˃X2)
PGPE250	12.47 ± 2.85 ^b^	1.20	1 (˃X2)
PGPE500	11.89 ± 3.87 ^b^	1.01	1 (˃X2)

^a^ *p* = 0.000 compared with the control group, ^b^ *p* = 0.017 compared with the CLP group, one-way ANOVA-Tukey test.

## Data Availability

Original data supporting the findings of this study are available. No copyright permissions are required for the figures in this study.
